# Microfluidic Surface Plasmon Resonance Sensors: From Principles to Point-of-Care Applications

**DOI:** 10.3390/s16081175

**Published:** 2016-07-27

**Authors:** Da-Shin Wang, Shih-Kang Fan

**Affiliations:** Department of Mechanical Engineering, National Taiwan University, Taipei 10617, Taiwan; amydsw@gmail.com

**Keywords:** surface plasmon resonance (SPR), microfluidics, immunosensing

## Abstract

Surface plasmon resonance (SPR) is a label-free, highly-sensitive, and real-time sensing technique. Conventional SPR sensors, which involve a planar thin gold film, have been widely exploited in biosensing; various miniaturized formats have been devised for portability purposes. Another type of SPR sensor which utilizes localized SPR (LSPR), is based on metal nanostructures with surface plasmon modes at the structural interface. The resonance condition is sensitive to the refractive index change of the local medium. The principles of these two types of SPR sensors are reviewed and their integration with microfluidic platforms is described. Further applications of microfluidic SPR sensors to point-of-care (POC) diagnostics are discussed.

## 1. Introduction

A striking property of a metal surface is the collective oscillation mode of conduction electrons termed surface plasmons [1,2]. Surface plasmons are present in many metals with various surface geometries, from planar-metal surfaces to metal nanostructures [3,4,5]. Due to the resonance with surface plasmons, the local electromagnetic wave is selectively magnified, which in turn intensifies many optical phenomena at the surface plasmon frequency, such as absorption, scattering, transmission [6,7], and photoluminescence [8,9]. The drastic optical effects all arise from the local field enhancement at the interface, which extends to hundreds of nanometers [10,11]. With advances in the fabrication and manipulation of materials at the nanometer scale, the surface plasmon modes arising from various metal nanostructures have been subject to intense investigations in recent years. The surface plasmon resonance (SPR) is of great value in biomedical applications, being primarily utilized in a biosensor because the resonance conditions of surface plasmons are very sensitive to the dielectric property at the interface, which is modified upon binding with biomolecules [12,13,14,15]. The SPR sensor using a planar thin gold film has been widely exploited in biomedical research and integrated as commercial systems by companies such as BIAcore (Uppsala, Sweden), now acquired by GE healthcare (Chicago, IL, USA); the SPR sensor provides a label-free, real-time, and high-throughput analysis of biomolecular interaction. The commercial BIAcore systems have been widely adopted by academia and pharmaceutical companies for the use in drug discovery, antibody characterization, proteomics, immunogenicity, biotherapeutic development and manufacture, and many life science research applications. More versatile and easy SPR biosensing using gold nanoparticles has been undertaken in many research laboratories [16]. Other sensing techniques such as surface enhanced Raman scattering (SERS) also make use of plasmonic substrates to magnify its signal. As a biosensor, SPR sensor has the advantage of a non-labeling technique, which is a strength over other conventional techniques such as fluorescence and isotope labeling; this advantage has another even more promising feature—real-time observation of the binding event and determination of analyte concentrations and binding constants [17]. Since its use in biosensing, the SPR biosensor has been implemented into several formats from fiber optics sensors to miniaturized sensors for point-of-care (POC) diagnostics [18,19]. In this review, the fundamentals of surface plasmon resonance and the setup of a typical SPR sensor are first introduced. Recent developments integrating microfluidic techniques on SPR sensing platforms to make POC devices are then briefly reviewed. Finally, the problems and prospects for a microfluidic SPR sensor to be used in POC practice is discussed.

## 2. Working Principles of Surface Plasmon Resonance Sensor

### 2.1. Planar Surface Plasmon Resonance

**Figure 1:**
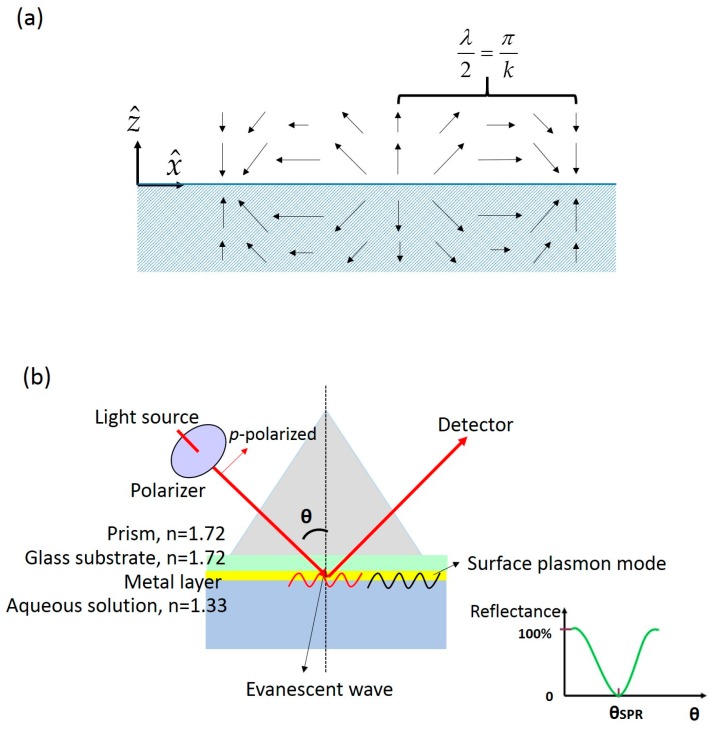
(**a**) A schematic illustration of the electrical field distribution of the surface plasmon at planar metal-dielectric interface; (**b**) the experimental setup of a typical surface plasmon resonance sensor in which a prism is used to produce the evanescent wave.

A surface plasmon is characterized by an electrical field that oscillates along the metal dielectric interface but decays exponentially in the direction perpendicular to the interface. The actual form of the SP electrical field is [20,21]:

(1) $$\vec{E}=E_{0}(\hat{x}+i\hat{z})e^{i(kx-\omega t)}e^{-k\left|z\right|}$$

where *E*_0_, *k*, ω are the amplititude, the wave vector, and the angular optical frequency of the electrical field, respectively. $\hat{x}$ and $\hat{z}$ are unit vectors as shown in the Figure 1a. A schematic illustration of the SP electrical field described by Equation (1) is shown in Figure 1a. When a *p*-polarized beam undergoes total internal reflection from a prism (denser medium) to an aqueous solution as shown in Figure 1b, there exists an evanescent electrical field (see Equation (2)) along the interface [22], which has exactly the same form as the SP as Equation (1):

(2) $$\vec{E}=E_{0}(\hat{x}+i\hat{z})e^{i(k_{t}x\sin\theta \cdot \frac{n_{i}}{n_{t}}-\omega t)}e^{-\beta \left|z\right|}$$

where β=kt(sin2θ⋅ni2nt2−1)12, *k_t_* is the wave vector in the transmitted medium, θ is the incident angle, and *n_i_*, *n_t_* are the refractive indices of the incident and transmitted medium, which are a prism and an aqueous medium in the setup of a typical SPR sensor. A typical SPR sensor is a metal thin film facing the sensing medium as shown in Figure 1b. The evanescent field can couple to the SP mode and excite the surface plasmons when the wave vector and frequency of the evanescent field match that of the SP. The wave vector of surface plasmon is:

(3) $$k_{sp}=\frac{\omega }{c}\sqrt{\frac{\varepsilon_{m}n^{2}}{\varepsilon_{m}+n^{2}}}$$

where ε_m_ is the dielectric constant of the metal film, *n* is the refractive index of the adjacent medium, ω is the angular optical frequency, and *c* is the speed of light in vacuum. The corresponding wave vector of the evanescent field along the surface is $k_{t}\sin\theta n_{i}/n_{t}$; surface plasmons with the wave vector equal to $k_{t}\sin\theta n_{i}/n_{t}$ will be excited and the excitation is observable on monitoring the reflectance. At light frequency ω, there is a unique *k* for surface plasmons according to the dispersion relation [23]; when light of a specific ω is used to generate surface plasmon resonance, it requires a match of the wave vector of the evanescent field with the SP wave vector as shown in Figure 1b. The wave vector of the evanescent field can be adjusted to match that of the SP by tuning the incident angle. The excitation occurs at a specific angle where a dip in the reflectance appears. This method allows accurate measurement of the frequency and wave vector of the surface plasmons; this excitation configuration is called the Kretschmann configuration [23]. The surface plasmon resonance is characterized simply by a resonance angle θ_SPR_. As the SPR condition shifts in response to the variation of dielectric constant of the local medium, the shift of resonance condition is detected as a shift in the resonance angle. SPR angle spectra is the SPR characteristic widely used in chemical and biological sensing.

### 2.2. Basic Setup of Planar Surface Plasmon Resonance Sensor

**Figure 2:**
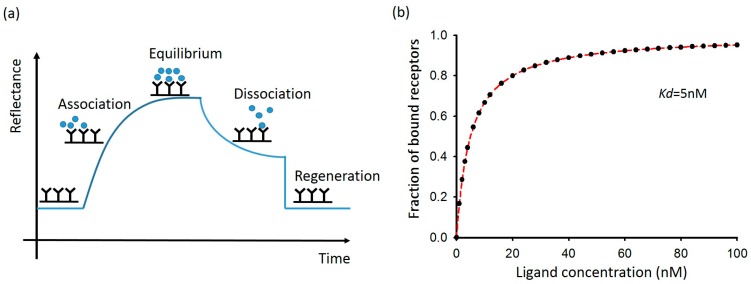
The kinetic and equilibrium state of a receptor and ligand binding event measured by an SPR sensor. (**a**) Reflectance of an SPR sensor when a binding event occurs, attains equilibrium, dissociates, and eventually returns to the original state; (**b**) The equilibrium of binding at various ligand concentrations determined by an SPR sensor.

To utilize the evanescent wave in exciting SPR, light must be subject to total internal reflection (TIR), which requires the light to travel in a path from a denser medium to a less dense medium. The components of the setup comprise a prism, a polarizer, a slide coated with a metal film, and a cell loaded with a liquid sample as shown in Figure 1b. The polarizer serves to select *p*-polarized light that generates an angle-modulated evanescent field as indicated in Equation (2). The metal film is thin (~50 nm) within the penetration depth of the evanescent wave so the evanescent field can excite the surface plasmons at the metal and aqueous interface. The incident angle of light is tunable with a motor and the angle spectrum reveals the condition at which surface plasmon resonance occurs. The angle spectra can be theoretically estimated by electromagnetic Fresnel equation [22] as long as the dielectric constant and thickness of each layer are known. Test molecules in an aqueous solution bind to the metal surface and their effects resemble that of adding an optical thin layer with an effective dielectric constant. The resonance angle is thus shifted accordingly. From the measurement of the angle shift, the test sample is detected. A typical experimental setup has a fixed incident angle at a position near the resonance angle; instead of angle, the reflective intensity is recorded as shown in Figure 2a. The binding of a test sample, dissociation of attached molecules, and a regeneration of the surface are all indicated as the reflectance changes. The results of an SPR experiment convey great information; the kinetic curve detected through reflectance enables the user to measure the association and dissociation of a biomolecular complex within a given time span; serial kinetic curves allow the user to determine the affinity and equilibrium constant of a biomolecular binding event. A detailed description of the mathematical treatment using SPR signals can be found elsewhere [24]. In short, for a ligand-receptor binding reaction, the equilibrium constant *K_d_* can be decided by finding the fraction of bound receptor molecules at various concentrations of ligands. The mathematical relation is expressed in Equation (4):

(4) $$receptor\;and\;ligand\;binding:\;R+L\to RL\;fraction\;of\;bound\;receptor\;molecules=\frac{\left[RL\right]}{\left[R_{t}\right]}=\frac{\left[L\right]}{K_{d}+\left[L\right]}$$

where [*R_t_*] and [*RL*] are the concentrations of total receptors and receptor-ligand complexes, respectively; [*L*] represents the ligand concentration in the solution.

As the SPR signal is linear to the quantities of the bound ligands, which form complexes with receptors, the SPR signal can be a direct indicator of [*RL*], and the SPR signal for [*R_t_*] is the maximum signal obtained with increasing ligand concentration [*L*] [24]. The equilibrium constant *K_d_* can be thus acquired by fitting the plot (Figure 2b). A typical SPR sensor works in this manner.

For high-throughput SPR sensing, an array of hundreds of biomolecule spots are made by a glass syringe or inkjet printing on the gold film [25,26,27,28]. An objective lens is placed in front of the detector to capture the image of these spots. The reflectance (SPR signals) of each spot is simultaneously read from the image; a scanning stage is equipped to hold and scan the whole sensor. The incorporation of a prism to excite surface plasmons is currently the most common design of commercial SPR sensors. Other SPR sensor formats for miniaturization and flexibility have been devised to replace the bulky setup of a prism, but the prism-based SPR sensor remains the most stable and sensitive design.

### 2.3. Other Formats of Planar SPR Sensors

**Figure 3:**
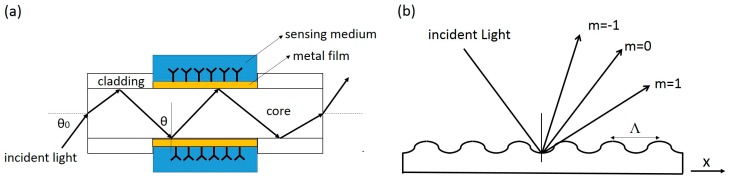
Schematic illustrations of (**a**) a fiber-optic SPR sensor and (**b**) light diffraction upon a periodic grating.

As explained above, surface plasmons are typically excited with an evanescent wave accompanying total internal reflection. Using a prism is just one method to produce evanescent waves but it has disadvantages such as large size and high cost. An optical fiber is a promising alternative to a prism because light also propagates in total internal reflection within the fiber, and a fiber can be readily adapted for miniaturization and remote sensing, requiring no mechanical adjustment of parts [29,30]. In a fiber-optic SPR sensor, the cladding layer of fiber is removed and a thin layer of metal film is coated on the core. The metal film faces out in contact with an outer medium, which can be liquid medium or air. The metal film surface is immobilized with some functional molecules to capture the target analyte (see Figure 3a). The evanescent wave generated by the total internal reflection (θ > critical angle) from the core to the medium excites the surface plasmons at the metal layer and the medium interface. As the light enters the fiber at a fixed angle θ_0_, for monochromatic light, the excitation of surface plasmons results in an attenuation of transmitted light; the change of SPR conditions due to analyte binding hence exhibits a variation in the loss of transmitted light. For polychromatic incident light, the SPR causes a characteristic dip in the spectrum of transmitted light. The main challenge of a fiber-optic SPR sensor is the unstable performance due to the deformation of the fiber; usually, the sensitivity is generally reduced in response to deformation. Prism-based SPR sensors generally have superior performance in sensitivity (10^−6^ refractive index unit change [31]), but a fiber-optic SPR sensor possesses great potentials for miniaturization. It has been integrated into a smartphone using the LED flash as the incident light and the camera on the phone as the detection unit [32]. It appears to be the best SPR format for flexible adaptation and miniaturization [33].

Another promising format of SPR sensor, grating-based SPR sensor, does not employ total internal reflection and evanescent wave, but makes use of the light diffraction property of a grating. Basically, a monochromatic and polarized light incident on a periodic grating is split into a series of beams as shown in Figure 3b, the wave vector in the x-direction is modulated by the grating structure and the ambient medium according to Equation (5):

(5) $$k_{mx}=\frac{\omega }{c}n\sin\theta +m\frac{2\pi }{\Lambda }$$

where ω is the angular optical frequency of the incident light, c is the speed of light, n is the refractive index of the medium, θ is the incident angle, and Λ is the grating period. When the wave vector in the x direction matches the wave vector of surface plasmons as indicated in Equation (3), a resonance occurs for different order m. A scan of angle near certain order of the reflected light shows a dip in reflectance, and the angular spectrum is very sensitive to the local refractive index n [34,35]. Light exciting the surface plasmons illuminates the surface from the sample side, allowing better imaging of the chip. Such a grating is also easily fabricated and appropriate for mass production.

### 2.4. Localized Surface Plasmon Resonance Sensor

Aside from the typical SPR sensor, which is simply a thin metal layer with surface plasmon modes propagating along the interface, other metal nanostructures carry confined SPR modes that can also be utilized for sensing, as the frequency of the localized SPR mode is very sensitive to the interfacial refractive index change. Sensors utilizing closed metal nanostructures are called localized SPR (LSPR) sensors. One common example is the gold nanoparticle, which absorbs light at a specific SPR frequency. The surface plasmon resonance in metal nanoparticles were first described primarily by a classical idea, i.e., solving the Maxwell’s equation with some given boundary conditions. With an approximate estimate of dielectric constant *ε* of the particle, we can calculate the reflection and extinction of an electromagnetic field at the surface with a given geometry. One notable example is the Mie calculation [36,37], which solved the extinction and scattering of a plane electromagnetic wave on a spherical particle. This calculation is performed on solving Maxwell’s equation in spherical coordinate with a boundary condition that defines a sharp discontinuity at the surface of a spherical particle of radius R. According to Mie’s results [38], the extinction cross section of a small particle is:

(6) σext(ω)=9ωcεm32Vε2(ω)[ε1(ω)+2εm]2+ε22(ω)

where *V* is the particle volume; ε_m_ and ε_1_ + *i*ε_2_ are the dielectric constants of the medium and the particle, respectively. According to Equation (6), the extinction of light depends on frequency, with a maximum occurred when ε_1_ *(*ω*) =* −2ε_m_, at which frequency much light is converted into the surface plasmons of the metal particle.

**Figure 4:**
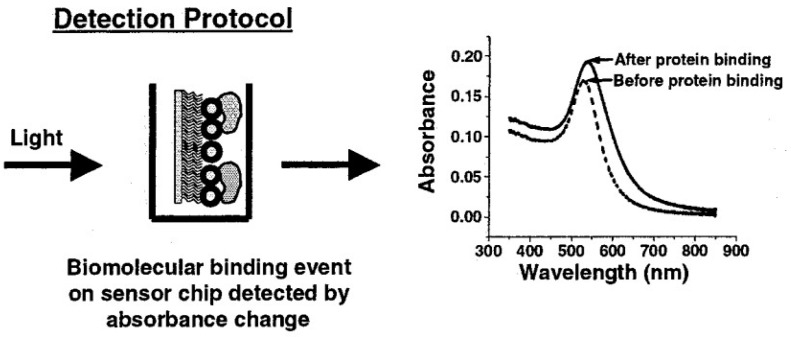
The detection protocol of a LSPR sensor. Here the LSPR sensor is a layer of gold nanoparticles immobilized by self-assemble silane monolayer on the glass substrate. Metal nanostructures other than nanoparticles can work in a similar manner. The binding of analytes on a LSPR sensor results in a shift of the absorption spectrum as well as an increase in peak amplitude. Reprinted with permission from [39]. Copyright 2002 American Chemical Society.

Mie’s calculations successfully explain the observed extinction spectrum of the dilute metal particle solution. Mie’s results also indicate the feasibility of using metal particles as sensing devices through the dependence of SPR maximum on the ambient dielectric property, ε_m_. The SPR peak frequency depends also on the particle size because ε_1_ (ω) varies for particles with different sizes. The SPR peak frequency can hence serve as a sensing signal, provided that the binding of analytes modifies the ambient refractive index or causes the particles to form clusters. The typical detection protocol of a LSPR sensor is illustrated in Figure 4. The absorption spectrum is taken from the LSPR sensor surface before and after the binding of analytes. The binding of analytes generates local refractive index changes at the interface, resulting in a shift of the absorption spectrum [39,40]. Other mechanism for sensing is to cause the metal nanoparticles (work as LSPR sensors) to aggregate upon the addition of a test sample. For example, in the case of immunosensing, antibodies are first conjugated on the surface of metal nanoparticles; the binding of antibodies onto the test sample antigens causes the metal nanoparticles to form aggregates, and thereby generates a shift of resonance frequency that can be detected using the same protocol shown in Figure 4 [41,42,43]. Not only nanoparticles but also many metallic nanostructures exhibit the same properties, and can thus serve as biosensors [44,45].

### 2.5. Limitation of Surface Plasmon Resonance Sensor

**Figure 5:**
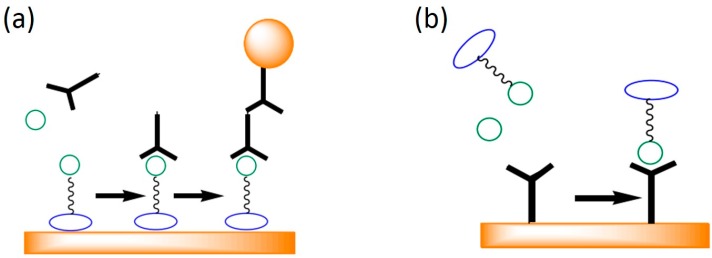
Special designs of SPR sensors for small molecule detection. (**a**) Primary antibodies and second antibodies conjugated with gold nanoparticle are added to enhance the signals; (**b**) A competitive immunoassay where antigens labelled with high mass molecules are used to compete with free analytes [48].

Surface plasmon resonance sensing is a label-free technique. No probes are labeled on analytes and the SPR signal changes solely come from the local refractive index changes induced by the binding of analytes. The refractive index changes are proportional to the mass loaded on the sensor surface; therefore, smaller molecules generate lower signals and pose challenges for SPR sensing. The smallest detectable molecular weight by SPR sensors was reported to be ~180 Da [46,47]. To attain optimal sensitivity, specific protocols are designed for SPR detection of small molecules. These designs involve the use of a second molecule with labels or a competitive assay with large molecules. These two schemes are illustrated in Figure 5 [48]. In Figure 5a, analytes are captured on the sensor surface, and then the primary antibodies are attached to provide the required mass; second antibodies conjugated with gold nanoparticles can be added to further enhance the signals. In Figure 5b, previously prepared antigens labeled with high mass molecules are used in competition with the unlabeled sample analytes. The SPR signal in this competitive immunoassay is inversely proportional to the analyte concentration. For small molecules detection, special designs of surface chemistry and the addition of labeled molecules are required for SPR sensors.

## 3. Microfluidic Surface Plasmon Resonance Sensor

Integration of SPR sensing technology with microfluidics offers the advantages of automation, small volumes, rapid processing, and perhaps enhanced sensing efficiency with a proper design [49,50,51]. Microfluidics might increase the reaction rate, reduce the diffusion time, and regenerate the surface more efficiently [52]. The automation through microfluidics gives improved reproducibility and increasingly precise control over each reaction stage [53]. The integration of sensing and microfluidic system is important to attain a high level of performance. This is particularly true for parallel SPR imaging as the sample simultaneously reacts with multiple ligands immobilized on a single chip, in which microfluidics can help to increase the throughput of a single chip and provide better delivery of the sample flow.

### 3.1. Flow-through SPR Sensor

**Figure 6:**
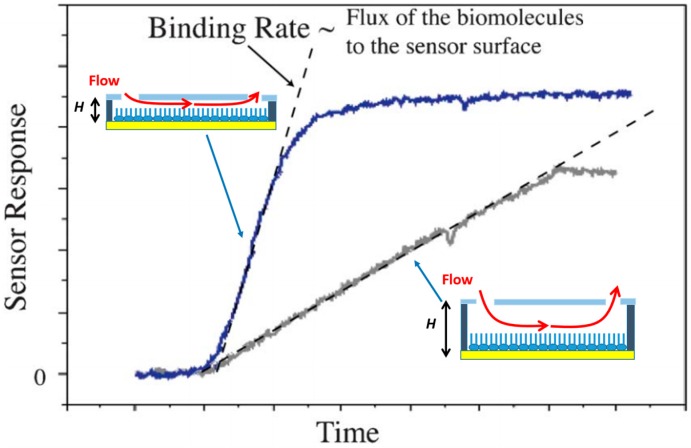
Typical sensor response vs. time; the blue and grey data sets represent higher and lower flux of target analyte, respectively. The slope of the time-series response is proportional to the flux of analyte to the sensor surface. Reprinted with permission from [57]. Copyright 2013 The Royal Society of Chemistry.

Applications in proteomics and drug discovery demand screening of many analytes from a single SPR imaging experiment. The SPR imaging sensor is typically integrated with continuous flow-through channels. The Biacore FLEXChip system serves for the purpose with a 400-spot chip housed on a flow cell with volume only 46 µL required for a single sample. Most commercial systems interrogate only a single sample on an arrayed chip, which requires multiple samples to be tested in a serial manner and hence time-consuming. Ouelle et al. [54] designed a 264 element-addressable chambers for up to six analyte inputs; each chamber had a 700 pL volume, connected with a serial diluted network that allowed simultaneous interrogation of a sample at varied concentrations. The device consisted of two polydimethylsiloxane (PDMS) layers; the lower layer had channels to deliver the analyte flow, and the upper layer contained 1132 microvalves to isolate and control the cross flow from multiple inputs into an individual chamber. As the SPR signal is sensitive to the temperature variation, Lee et al. [55] designed a simpler multiplexed SPR sensor for multiple analytes with temperature control. The microfluidic chip consisted of three PDMS layers and a glass substrate. The first layer contained micropumps and microvalves to deliver the analyte flow; the second layer was made as a microchannel array in which the sample was delivered; the third layer was an array on which each ligand was immobilized. The bottom glass substrate was fabricated with micromachined heaters, temperature sensors, and flow sensors. Multiple analytes flowed through a one-dimensional channel array for multiple ligand tests. The thermal noise was reduced; the SPR sensing had high sensitivity and stability. Another critical problem encountered in SPR sensing is that nonspecific adsorption causes substantial signal changes; therefore, an appropriate choice of a reference is important for the subsequent calculations of kinetic parameters. Grasso et al. [56] designed a Y-shape flow cell in which solutions with and without analytes were simultaneously eluted through the Y-shape channel. As two fluids flowed in a laminar mode within the same microchannel, one side served for measurement of the SPR signal and the other side offered an in-line reference. This device showed how integrating an SPR sensor with a microfluidic system solved a fundamental problem in SPR measurement. Lynn et al. [57] investigated the optimal geometric and operational design of a flow cell in the delivery of analyte flux. An increased analyte flux enhanced the analyte amounts captured on substrate in a given period. A larger signal was produced in a brief interval as shown in Figure 6. This consideration applies only for the axial flow without active and passive mixing. They have predicted a scaling law that suggests the analyte flux (*J*) has the specific relation with channel height (*H*), *J ≈ H^−^*^2/3^ for systems with a constant volumetric flow rate, and *J ≈ H^−^*^1/3^ for systems with a constant average fluid velocity. As the channel height (*H*) decreases, the analyte flux increases and improves the analyte capture at a given time, thus increasing the LOD (limit of detection) of the SPR sensor.

The flow-through cell has also been implemented on a waveguide-based SPR sensor for miniaturization. Krupin et al. [58] designed a flow-through microfluidic channel etched into the top of waveguide cladding. The sensing region consisted of gold stripes (width 5 μm, thickness 22 nm) embedded in polymer. Bulk medium sensing was attained on sequentially injecting varied test solutions. Selective detection of cells and proteins was achieved by functionalizing the gold strip with appropriate antibodies. The protein sensing was demonstrated using bovine serum albumin (BSA) and the LOD was 12 pg/mm^2^, about ten times that of a conventional benchtop SPR sensor (LOD ~ 1 pg/mm^2^) [59]. Although less sensitive than a conventional prism-based sensor, the design is portable, easily and cheaply fabricated and easily adaptable for POC applications. Besides the waveguide format, Dostalek et al. [60] demonstrated a microfluidic grating-based SPR sensor in which a glass flow cell chamber was sealed on an array of miniaturized diffraction gratings; each grating in the array served as an independent sensing element for a specific target. The designed microfluidic grating sensor has been shown to successfully detect the refractive index change of the ambient medium, but the detection of biomolecular binding has not been realized on a grating-based microfluidic SPR sensor.

LSPR sensors of various nanostructures by themselves are miniaturized versions of SPR sensors that require neither a prism nor complex optical systems, merely a simple measurement of transmittance. Zhou et al. [61] fabricated nanocrescent arrays and used the arrays as a plasmonic sensor in which a slight refractive index change results in the resonance frequency shift or transmittance change when monitored at a constant frequency. This nanocrescent plasmonic sensor was further integrated with a microfluidic system in which two inlets, one delivering sodium chloride solution and the other infusing deionized water at varied flow rates, created sodium chloride solutions of various surface concentrations at the detection region. The microfluidic design with two inlets adjusting concentration gradients at the detection region readily offered a standard reference curve for the sensor.

Compared to a prism-based SPR sensor explained in Section 2.2, the LSPR sensor using nanostructure arrays appears to be more portable than a prism-based SPR sensor explained in Section 2.2, and is easily integrated with microfluidics [55,62]. As the LSPR sensor typically employs a spectrometer to detect the absorbance of SPR mode, the material for microfluidic cell must have low absorbance at wavelength 450–700 nm, as in this region most nanoparticles absorb light. Huang et al. [63] chose cyclic olefin copolymer (COC) for its biological and micro-process compatibility and created a microstructure on it by injection molding. The flow cell was designed to have a diamond shape to avoid the formation of air bubbles. Two flow cells were built in parallel; one cell with no nanoparticles on it served as a blank. For multiplexed sensing, He et al. [64] introduce patterned 7-channel nanoparticle arrays made with hole mask colloidal lithography and sealed with PDMS microfluidic channels. The fabrication method was a simple, inexpensive, and scalable technique. Streptavidin at varied concentrations was added in each channel embedded with biotinylated nanoparticle arrays; the 7-point dynamic binding curve was acquired by measuring transmittance with a standard UV–vis spectrometer. The 7-channel arrays were extended to 96 spots for parallel protein sensing.

### 3.2. Droplet-Based SPR Sensor

Most prism-based and nanostructure SPR sensors are equipped with flow-through cells to deliver the analytes. This type of measurement usually carries a substantial dead volume and requires a relatively large amount of samples; a droplet-based system can effectively reduce the required volume of samples. This benefit arises particularly when the analyte is a precious protein. Yin et al. [65] demonstrated the SPR measurement of IgG and anti-IgG binding kinetics using a microdroplet of sub-μL volume. The amount of IgG in the sample volume was only 0.04 µg, 1/7000 times of the sample for a conventional flow-through SPR sensor. A microsyringe (1 μL) with precision 0.02 μL was used to dispense the analyte droplet onto the surface. A CCD camera with an objective lens captured the image of the droplet and recorded the reflected intensity. The central region of the droplet was circled for the intensity recording. A time-dependent intensity revealed the binding kinetics. A key issue in the kinetic measurement is that the droplet cannot dry out during the measurement; the volume change arising from droplet evaporation does not affect the binding and signal detection, but the reduction in volume increases the salt concentration in the droplet, which can affect the SPR signal since it can cause a change of the local refractive index. Deionized water was hence filled within the gap in the small chamber to maintain the humidity. The droplet-based system produces equilibrium constants *K_d_* comparable with that of a conventional flow through system but requires a much smaller quantity of analyte.

**Figure 7:**
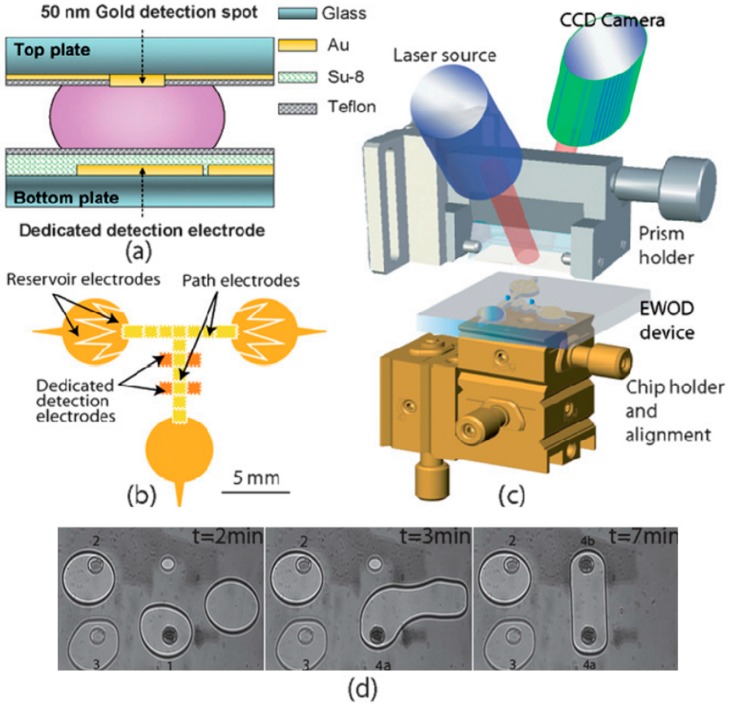
A schematic illustration of a EWOD-SPR sensor chip; (**a**) from side view, (**b**) from bottom view, (**c**) mounted on the SPR measurement system, (**d**) SPR imaging of four EWOD-SPR sensing spots. Reprinted with permission from [70]. Copyright 2009 The Royal Society of Chemistry.

As described earlier, multiple ligands are immobilized on an SPR sensor for high-throughput testing of a sample. Most commercial systems use a flow cell with only a single inlet and outlet, and are designed for a single stream of sample; multiple samples have hence to be tested sequentially. In a continuous flow system, attaining parallel testing of multiple samples on multiple ligands requires each ligand to be located in a microchamber; the microchambers are connected with crossed flow channels to deliver separate analytes. The crossed flow channels require multiple valves to control the desired flow at the channel intersection. The incorporation of such structures becomes increasingly complicated as the array size increases. An alternative to the continuous-flow system is digital microfluidics using electrowetting on dielectric (EWOD) [66,67,68,69]. EWOD is a phenomenon in which the dielectric surface property is modulated with an electrical voltage. The contact angle of a droplet can be selectively modified, which causes the droplet to move on the surface. As droplets are independently moved on addressable electrodes, EWOD appears to be an excellent technique to manipulate the analyte delivery on an SPR sensor. Malic et al. [70] introduced and proved the concept by testing varied solutions simultaneously at four SPR sensing spots. First of all, the experiment demonstrated the feasibility of obtaining SPR signals on an EWOD chip. The hydrophobic surface of the EWOD top plate was opened with a window coated with a gold film (50 nm) for SPR detection (Figure 7). The ligands tested were immobilized on the gold film window. Droplets of separate samples were retrieved from different reservoirs and then moved to the detection electrodes. The EWOD device was placed beneath the prism and a CCD camera is used as a detector to simultaneously record the SPR signals from four detection electrodes as shown in Figure 7d. Although only a few detection electrodes were fabricated to verify the concept, the flexibility and the precision to control a specific analyte droplet on a specific detection electrode were attractive features for the implementation of high-throughput SPR sensing. Besides EWOD fluidic control, the SPR sensing window on an EWOD upper plate can be easily modified for ligand testing or possible signal enhancement. Malic et al. [71] incorporated periodic gold nanospots on the gold layer of the EWOD sensing window, which doubled the signal on complementary DNA hybridization. The kinetic binding was measured at a stop-flow condition; the mixing with a buffer droplet was attained by applying a pulsed voltage on mixing electrodes. The reservoir of the solution required sample volume only 0.5 µL. A droplet (90 nL) was dragged from the reservoir and manipulated by the electrodes for the following mixing and detection. The results demonstrated the enhanced sensitivity of a nanostructured EWOD SPR imaging sensor with ultra-small sample volume required for DNA hybridization. Not only the final hybridization but also the preparation of DNA probe on the sensor surface for subsequent hybridization was accomplished with EWOD microfluidics [72,73]. The EWOD device covalently attached DNA probes on the SPR sensing electrode by dispensing, merging, mixing and separating droplets.

The DNA probe droplets were brought to the SPR sensing electrode for passive immobilization or applied with an electric potential for 2 h. The applied electric potential had improving effects in the density and orientation of immobilized probes, which increased the efficiency of the subsequent hybridization and resulted in enhanced SPR signals. The EWOD-SPR microfluidic sensor is capable of diluting DNA probes, independently and simultaneously immobilizing the probes on selected electrodes, optimizing the density and orientation on a sensor surface, and enabling real-time SPR sensing simultaneously at multiple spots.

### 3.3. Microfluidic Surface Plasmon Resonance Sensor for Point-of-Care (POC) Diagnostics

The features of an ideal POC device include fast turn-around time, ease of use for non-professionals, portability, robustness, and easy storage of the reagents. Microfluidics attains the automatic operation of mixing sample and reagent, cleaning and washing, and facilitating sensing. Among devices based on various microfluidic techniques, those with capillary flow devices are the most widely distributed and commercially successful forms of microfluidic POC devices [74,75]. The representative examples are lateral flow devices (pregnancy test strips) and glucose test kits. The materials used in these devices are paper and membrane, which attain the goal of low-cost and high-volume production, contributing to the prevalence of these devices. A pregnancy test strip is an example of LSPR sensor, in which gold nanoparticles are conjugated with antibodies to capture the analytes and aggregate to induce colorimetric changes. Tseng et al. [76] introduced a convenient, cheap, rapid, and efficient technique to prepare nanoparticles on a test paper for sensing. The test paper was first deposited with a thin gold film. The surface was then illuminated by excimer laser light and became liquefied through photothermal effects. The surface tension made the liquefied metal surface deform into spherical shapes during cooling and form nanoparticles on the paper. These nanoparticles formed on the paper provided an excellent paper-based microfluidic SPR biosensor, and had the advantages of being simple, cheap, easy to use, portable, disposable, and environmentally compatible, which is suitable for POC diagnostic purpose.

To apply SPR biosensor to POC diagnostics, the sensor material must be cost-effective and suitable for mass production. However, up till now, most microfluidic SPR sensors were fabricated with polydimethylsiloxane (PDMS) for fast prototyping at a small scale. Malic et al. [77] used hot embrossing techniques to mold fluidic and plasmonic features on a hard thermoplastic bottom and included active fluidic elements such as pneumatic valves on a top thermoplastic layer. The plasmonic features used for sensing were monolithically blazed nanograting structures covered with a gold film (50 nm), located in the chambers connected with multiple microfluidic channels. A transmission-based optical setup was used for multiplexed sensing; the sensitivity and specificity were examined for the label-free detection of soluble cell-surface glycoprotein sCD44. The instrument exhibited a sensitivity ranging from nanomolar to picomolar concentration, which is clinically relevant. The technique is simple, single-step, cost-effective, amenable to mass production and capable of producing miniaturized devices for POC diagnostics. Other cost effective materials such as polymethylmethacrylate (PMMA) have been used to fabricate SPR sensors. Tokel et al. [78] designed a portable, cheap, and disposable microfluidic SPR chip that detected and quantified the bacteria *Escherichia coli* (*E. coli*) and *Staphylococcus aureus*. A microchannel shape was cut in a layer of double-sided adhesive (DSA, thickness 50 μm) using laser cutter to create a channel with a volume of 4 µL; the DSA layer held together the PMMA layer and the gold-coated glass substrate. A single inlet and outlet port were opened on a PMMA layer in which the sample was injected into the channel and bound on the gold surface. The PMMA-DSA-glass (coated with gold) was then mounted on the prism for SPR measurement. The microfluidic SPR sensor was tested on the capture of *E. coli* with LOD of ~10^6^ CFU/mL. A lower LOD, ~10^5^ CFU/mL, with SPR has been reported [79], but it generally involves using a bulky SPR sensor with a more sophisticated design.

Non-labelling detection is a key advantage of SPR sensor, but this advantage, on the other hand, presents some challenges for POC applications of SPR sensor. The binding of analyte molecules induces a refractive index change at the interface and thus modifies the SPR condition. Not like labeling detection, only labeled analytes generate signals; other factors such as non-specific binding of molecules other than the analyte affect the SPR signals. Therefore, a pure sample containing few other molecules is preferable in SPR sensing. The preprocessing of a sample fluid, such as acquiring serum from whole blood, is essential in SPR sensing. For POC applications in which the entire process from sampling to sensing must be integrated on a chip, the preprocessing such as separation of blood cells from whole blood requires microfluidic elements not only to deliver but also to purify the sample. Microfluidic techniques used for separation, such as microcentrifuges, CD-type platforms [80,81], microporous filters [82,83], and designs utilizing inherent hydrodynamic separation [84] and active energy [85], are potential techniques to be incorporated in the miniaturized SPR sensors for POC application. Hemmi et al. [86] devised a CD-type microfluidic SPR sensor. The sample solution was driven by the centrifugal force to a grating-based SPR sensor on the CD platform. Reservoirs connected with microchannels [87] were fabricated on polydimethylsiloxane (PDMS) disk and then attached to a flat polycarbonate (PC) disk plate. A layer of Au-coated grating film was bonded on the PC disk for SPR sensing; white light was directed at normal incidence by an optical fiber and a spectrometer was implemented for the spectral measurement. The working principle of a CD-type platform is that the liquid in the reservoir located at a certain radius begins to overflow to the outermost reservoir at a specific rotation frequency. Hemmi et al. [86] demonstrated the immunoassay of IgA on the CD-type SPR sensor. The antibody solution, blocking reagent, sample, and the washing solution were dispensed from separate reservoirs; with rotation applied at varied frequency, the solutions flowed into the SPR detection chamber at the outermost reservoir in a sequence. The SPR spectral shift induced by each step—i.e., antibody adsorption, non-specific blocking, sample binding, and washing, was clearly observed. One shortcoming of this CD-type platform is the lower sensitivity of the incorporated grating SPR sensor relative to a conventional prism-based SPR sensor. However, the CD-type platform offers the potential of sample preprocessing, which eliminates the noise caused by non-specific adsorption from a complicated fluid such as whole blood. For POC applications, the sample is typically a small volume of blood from a finger prick (10~100 µL), which is not ready for the SPR measurement due to the non-specific adsorption. The CD-type microfluidic devices were shown [88,89,90,91] to be capable of separating plasma from whole blood and extracting DNA from blood. With the preprocessing integrated in the SPR sensor, an all-in-one SPR sensing system for POC is accomplishable. Table 1 summarizes the efficiency of various microfluidic SPR sensors in terms of detection limits, required volume, and analysis time.

## 4. Conclusions

Surface plasmon resonance is a fast, label-free, and sensitive technique for biosensing. It eliminates the troublesome procedures and cost of optical probe labeling. A conventional SPR sensor is a planar metal film deposited on a glass substrate and surface plasmon is excited using the Kretschmann configuration, which involves prism-based excitation. Various other formats have been designed to replace the bulky prism-based excitation; these formats include optical fiber and grating-based excitation methods. Besides planar metal films, metal nanostructures exhibit structure-dependent surface plasmon resonance; the resonance condition is sensitive to the dielectric constants of the metal and local medium. Changes induced by binding of analytes on the structural interface cause a frequency shift of surface plasmon resonance; this shift serves for sensing. The use of nanostructures for sensing further reduces the size of a sensor, realizing the purpose of miniaturization in a POC context. Microfluidics reduces the reaction volume and offers an automatic way to deliver the sample, wash, and regenerate the surface. Most SPR sensors are equipped with flow-through microfluidic systems; sophisticated designs of microchambers connected with multiple microchannels have been realized for a high-throughput SPR imaging sensor, in which multiple samples can be tested on multiple ligands immobilized on the sensor surface. Optimal designs of the channel dimension affect the sample flux and result in an enhanced signal. Droplet based microfluidic delivery has been implemented in an SPR sensor system to further reduce the sample volume, which is valuable when the analyte sample is scarce and precious. SPR sensing has also been carried out on an EWOD chip by selectively incorporating an SPR sensing window on the chip. Droplet actuation using EWOD can simultaneously and independently deliver multiple samples for SPR sensing at multiple spots, which exhibits the potential of using EWOD as a microfluidic means for the high-throughput SPR imaging sensor. Sample delivery using a capillary membrane or paper typically accompanies localized SPR sensors and appears to be a good choice for POC applications. Besides cellulose membrane and paper, thermoplastic material has been used as a substrate for the fabrication of microfluidic features and plasmonic structures, and is proposed as an economic choice for mass production and practical applications. As non-specific binding from molecules other than the analyte in a sample can contribute to significant SPR signals, sample preprocessing is an essential step to eliminate unwanted SPR signals and artifacts. Microfluidic components for separation and extraction on, for example, a CD-type centrifugal platform, are crucial to obtain pure samples for subsequent high-quality SPR sensing. In conclusion, various formats of SPR sensors from prism-based to fiber-optic SPR sensors have been devised to detect various biomolecules. Integration with microfluidics improves the efficiency of high-throughput SPR imaging sensors, enhances the SPR signals, and reduces the size of the system for POC applications. Future incorporation of advanced microfluidic components for separation and extraction may fully equip SPR sensors with the capability to work as an all-in-one POC diagnostic device.

## Figures and Tables

**Table 1:** Summary of various microfluidic SPR sensor formats.

SPR Sensor Type	Microfludic Formats	Detection Limits	Required Volume	Analysis Time	Refs.
Prism-based SPR sensor	Flow-through cell	0.2 μg/mL	100–1000 μL	5–20 min	[54,55,56,57,78]
	Digital microfluidic (EWOD, droplet-based)	1 μg/mL	0.2–1 μL	1 min	[65,70,71,72,73]
Waveguide, fiber-optic SPR sensor	Flow-through cell	100 μg/mL	~200 μL	10 min	[58]
Grating-based SPR sensor	Flow-through cell	100 μg/mL	---	10 min	[60]
	CD-based	200 μg/mL	20–40 μL	5 min	[86]
Localized SPR sensor using nanostructures	Capillary-driven (paper and membrane based)	---	---	---	[76]
	Flow-through cell	0.3–1 μg/mL	30–200 μL	10 min	[63,64]
